# Postoperative Vaginal Cuff Healing After Minimally Invasive Surgery for Endometrial Cancer: The Role of Postoperative Immunonutrition

**DOI:** 10.3390/jcm15062248

**Published:** 2026-03-16

**Authors:** Sevda Bas, Büşra Asena Torun, Eda Koyuncuoğlu, Oğuz Uyar, Tuba Yar, Seda Yüksel Şimşek, Sevtap Seyfettinoğlu, Mehmet Ali Narin

**Affiliations:** 1 Department of Gynecologic Oncology, Adana City Training and Research Hospital, University of Health Sciences, Adana 01120, Türkiye; b.asena.torun@gmail.com (B.A.T.); ekyncgl@gmail.com (E.K.); dr.oguzuyar@gmail.com (O.U.); tubaakarr@gmail.com (T.Y.); dryukselseda@hotmail.com (S.Y.Ş.); sevtaponcul@gmail.com (S.S.); mali_narin@yahoo.com (M.A.N.); 2 Department of Translational Medicine, Institute of Health Sciences, Cukurova University, Adana 01330, Türkiye

**Keywords:** endometrial neoplasms, laparoscopy, hysterectomy, wound healing, perioperative care, nutritional support

## Abstract

**Background**: This study aimed to evaluate the impact of postoperative immunonutrition on vaginal cuff healing in well-nourished patients undergoing laparoscopic surgery for endometrial cancer. The secondary objective was to assess postoperative complications occurring within 30 days. **Methods**: This prospective observational cohort study included patients who underwent laparoscopic surgery for endometrial cancer. Patients receiving postoperative oral immune-modulating diets were compared with those managed with a standard postoperative diet. Vaginal cuff healing assessed at postoperative 4th and 6th weeks. Postoperative complications within 30 days were recorded prospectively. **Results**: A total of 131 patients were included [immunonutrition group, *n* = 69; control group, *n* = 62]. At the 4th postoperative week, complete vaginal cuff healing was observed in 84.1% of the immunonutrition group and 75.8% of the control group [*p* = 0.24]. By the 6th postoperative week, complete healing rates were comparable [96.6% vs. 93.1%, *p* = 0.43]. In multivariable analysis, vaginal cuff closure time was independently associated with delayed cuff healing [*p* = 0.02]. Postoperative morbidity did not differ between groups. **Conclusions**: Vaginal cuff healing after laparoscopic surgery for endometrial cancer was primarily influenced by surgical factors, particularly vaginal cuff closure time, rather than postoperative immunonutrition.

## 1. Introduction

Endometrial cancer is the most common gynecologic malignancy in developed countries and is primarily managed with surgical staging [1]. Laparoscopic surgery has become the preferred approach compared with laparotomy due to reduced postoperative morbidity, shorter hospital stay, and faster recovery [2].

Laparoscopic surgery offers particular advantages for patients with endometrial cancer, who frequently present with comorbidities such as obesity and diabetes that adversely affect wound healing [3,4]. However, vaginal cuff healing after laparoscopic hysterectomy has been hypothesized to be delayed compared with open abdominal hysterectomy, partly due to the use of electrosurgical energy during colpotomy and the laparoscopic view, which may raise concerns about taking full-thickness bites and potentially result in smaller suture bites than those taken during an open approach [5].

Complete vaginal cuff healing is essential to prevent local complications, including infection and dehiscence, particularly in patients who require adjuvant radiotherapy [RT]. Current guidelines recommend initiating adjuvant RT within 6 to 8 weeks after hysterectomy, as delays beyond 9 weeks are associated with poorer oncologic outcomes [6,7]. Therefore, factors influencing early vaginal cuff healing represent a clinically relevant concern in minimally invasive gynecologic oncology.

Immunonutrition (IMN), containing arginine, glutamine, omega-3 fatty acids, and nucleotides, has been shown to modulate immune responses, reduce postoperative inflammation, and decrease infectious complications in patients undergoing major abdominal and oncologic surgery. IMN also promotes collagen synthesis and connective tissue growth, suggesting a potential role in wound healing [8]. Based on this evidence, IMN is recommended by the European Society for Clinical Nutrition and Metabolism [ESPEN] for patients undergoing major gastrointestinal or head and neck surgery. IMN has also been proposed to support surgical wound healing; however, evidence regarding its role in gynecologic oncology—particularly following laparoscopic surgery—remains limited.

The primary objective of this prospective observational cohort study was to evaluate the effect of postoperative IMN on vaginal cuff healing in well-nourished patients undergoing laparoscopic surgery for endometrial cancer. We hypothesized that postoperative IMN would increase the likelihood of complete vaginal cuff healing at the postoperative 4th week compared with standard postoperative care. The secondary objective was to assess postoperative complications occurring within 30 days.

## 2. Materials and Methods

This prospective observational cohort study was conducted at the Department of Gynecologic Oncology, Adana City Hospital, University of Health Sciences, Türkiye, between January 2024 and October 2025. The study protocol was approved by the institutional ethics committee [approval no. 07/12/2023-2981], and written informed consent was obtained from all participants in accordance with the Declaration of Helsinki, including consent for the publication of anonymized clinical images.

Eligible patients underwent elective laparoscopic surgery for biopsy-proven cancer or endometrial intraepithelial neoplasia. Patients with suspected endometrial pathology without preoperative biopsy were also included. The sample size was determined by consecutively enrolling all patients who underwent laparoscopic surgery at the institution during the study period.

Postoperative oral immune-modulating diets (IMDs) were incorporated into institutional postoperative care and offered to all eligible patients. Patients who received IMDs constituted the immunonutrition (IMN) group, whereas patients who were managed with a standard postoperative diet constituted the control group. Exclusion criteria included chronic immunosuppressive conditions, prior pelvic radiotherapy, severe hepatic or renal disease, conversion to laparotomy, vaginal route cuff closure, withdrawal of consent, or failure to attend scheduled postoperative follow-up.

### 2.1. Surgery

All procedures were performed by three experienced gynecologic oncologists following a standardized protocol. Surgical staging consisted of laparoscopic hysterectomy with or without bilateral salpingo-oophorectomy, with sentinel lymph node mapping and lymphadenectomy when indicated.

A circular laparoscopic colpotomy was performed using monopolar energy [50 W]. Vaginal cuff closure was completed laparoscopically using the same absorbable barbed suture (V-Loc™ 90, 0; Medtronic, Minneapolis, MN, USA) in a continuous running fashion, with two to three back bites to secure the proximal end. This technique was applied uniformly to minimize procedural variability. Operative time, blood loss, and complications were recorded prospectively. Vaginal cuff length and uterine weight were measured intraoperatively.

### 2.2. Postoperative Interventions

All patients in both cohorts were managed according to a standardized Enhanced Recovery After Surgery [ERAS] protocol. The protocol included early mobilization, standardized multimodal analgesia, and routine prophylaxis against thromboembolism and infection.

Measures for surgical site infection prevention were implemented in accordance with ERAS principles and included standardized perioperative antibiotic prophylaxis, strict postoperative glycemic control targeting blood glucose levels below 180 mg/dL, preoperative vaginal preparation with povidone–iodine, meticulous surgical technique, maintenance of normothermia using an under-body forced-air warming device, and early postoperative recovery strategies. All patients were instructed to maintain pelvic rest for six weeks following surgery.

### 2.3. Enteral Nutritional Supplementation

The IMD regimen consisted of two commercially available enteral formulas: Impact^®^ and Resource^®^. Each 237 mL serving of Impact^®^ provided 4.2 g of arginine, 430 mg of nucleotides, and 1.1 g of omega-3 fatty acids [eicosapentaenoic and docosahexaenoic acids], yielding approximately 237 kcal per serving. Each 237 mL serving of Resource^®^ provided 5 g of glutamine and approximately 20 kcal.

Patients in the IMN group consumed one serving of Impact^®^ and three servings of Resource^®^ daily for seven postoperative days [total daily volume 948 mL], starting within 24 h after surgery. The prescribed supplementation was intended to provide immune-modulating substrates rather than full caloric replacement and was administered in addition to the standard postoperative hospital diet. The control group received standard postoperative diet alone.

### 2.4. Compliance and Tolerance

Adherence to the immune-modulating diet protocol was assessed during scheduled postoperative follow-up visits. Compliance was defined as the proportion of the prescribed regimen completed during the 7-day intervention period and was calculated based on daily intake diaries and verification by package counts. A day was considered compliant only if the full prescribed daily supplementation, consisting of one serving of Impact^®^ and three servings of Resource^®^ (total daily volume 948 mL), was consumed. Partial intake was not classified as compliant for the purpose of compliance calculation. Compliance was calculated using the following formula:

Compliance (%) = [number of days on which both supplements were fully consumed/7] × 100.

Tolerance was evaluated by recording any interruptions or discontinuations of immune-modulating diets, together with the documented reasons for non-use, including gastrointestinal adverse effects or palatability-related complaints.

### 2.5. Outcome Assessments

Baseline patient characteristics and comorbidities were recorded. Preoperative nutritional status was evaluated using the Nutritional Risk Screening 2002 [NRS-2002] [8]. Patients with an NRS score of ≥3 were defined as being at nutritional risk. Surgical site infection was assessed according to Centers for Disease Control and Prevention [CDC] criteria [9]. Wound evaluations were performed daily during hospitalization and at scheduled postoperative follow-up visits for up to 30 days. Infections requiring medical or surgical intervention were considered clinically significant.

### 2.6. Assessment of Vaginal Cuff Healing

Vaginal cuff healing was assessed at postoperative weeks 4 and 6 by a single blinded surgeon [BAT] using validated criteria [10,11]. Complete vaginal cuff healing was defined as complete mucosal approximation without separation, exposed suture material, or granulation tissue. Incomplete healing included any deviation from these criteria. Patients exhibiting incomplete healing at follow-up were reassessed at two-week intervals until complete healing was observed.

Vaginal cuff bleeding was defined as any postoperative bleeding originating from the vaginal cuff, either observed during examination or reported by the patient. Vaginal cuff hematoma was defined as any blood collection at the vaginal cuff detected by transvaginal ultrasonography. Vaginal cuff infection was diagnosed based on clinical signs and symptoms such as purulent vaginal discharge, induration, erythema, or tenderness of the vaginal cuff. Cuff dehiscence was defined as a full-thickness separation of the vaginal epithelium.

### 2.7. Statistical Analyses

Statistical analyses were performed using IBM SPSS version 26.0 [IBM Corp., Armonk, NY, USA]. Continuous variables were compared using the *t*-test or Mann–Whitney U test. Categorical variables were analyzed using the chi-square or Fisher’s exact test. Logistic regression analysis was performed to identify factors associated with complete vaginal cuff healing at postoperative week 4. Results are presented as odds ratios with 95% confidence intervals. A *p* Value < 0.05 was considered statistically significant.

## 3. Results

**Figure 1:**
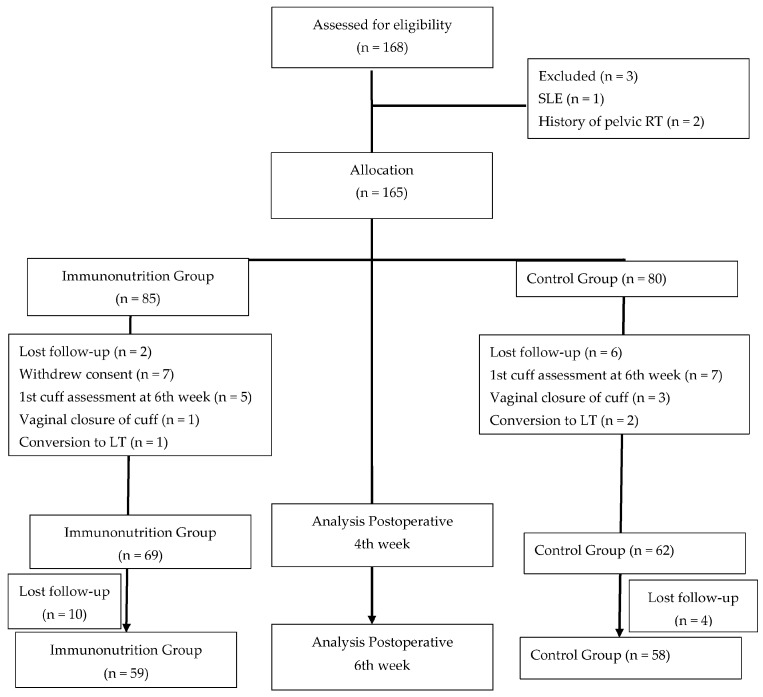
During the study period, 168 patients were scheduled for surgery at our clinic for a diagnosis or suspicion of endometrial cancer. One patient was excluded due to a diagnosis of systemic lupus erythematosus, and two patients were excluded because of a history of pelvic radiotherapy. Of the remaining patients, 85 who received postoperative immune-modulating diets were allocated to the immunonutrition group, while 80 patients were managed with a standard postoperative diet. Patient exclusions and losses follow-up at each stage are detailed in the flowchart.

The study flowchart and protocol variations are presented in Figure 1. Initially, vaginal cuff evaluations were planned for the 6th and 8th week after operation. However, the high rate of complete vaginal cuff healing observed at the 6th week prompted a protocol modification, and the first postoperative assessment was rescheduled to the postoperative 4th week in order to better capture early wound-healing differences. Consequently, 12 patients who had already undergone their first vaginal cuff evaluation at the 6th week were excluded from the final analysis to ensure methodological consistency. These 12 patients were comparable to the analyzed cohort, with no significant differences in baseline demographic, clinical, or surgical characteristics.

After exclusions, 131 patients were included in the 4th week analysis (62 control and 69 immunonutrition). For the 6th week evaluation, 117 patients were available. Baseline demographic and clinical characteristics of the study population are summarized in Table 1.

Baseline characteristics were comparable between groups. There were no significant differences in age, body mass index, smoking status, menopausal status, comorbidities, American Society of Anesthesiologists classification, or nutritional status. In the control group, seven patients had a history of breast or thyroid cancer, whereas in the IMN group, five patients had previously been treated for breast, thyroid, or colorectal cancer. All prior malignancies were clinically stable, and no patient had an active malignancy other than the current endometrial pathology at the time of enrollment. Among patients with a history of breast cancer, three patients in the control group were receiving selective estrogen receptor modulator and one was receiving an aromatase inhibitor, while one patient in the immunonutrition group was receiving selective estrogen receptor modulator during the perioperative period.

Surgical and pathological characteristics are presented in Table 2. Surgical and pathological characteristics were also similar between groups, including operative time, vaginal cuff closure time, cuff length, uterine weight, surgical procedure, histological subtype, and FIGO stage.

### 3.1. Postoperative Morbidity

Postoperative complications are summarized in Table 3. No significant differences were observed between the groups regarding febrile morbidity, readmission rates, or surgical site infection. In the IMN group, one patient developed pneumonia during postoperative hospitalization and received antibiotic treatment. In the control group, two patients developed urinary tract infections after discharge; one had a urine culture positive for *Pseudomonas aeruginosa*, while the other exhibited culture findings consistent with contamination. Both were managed with outpatient antibiotic therapy. Superficial incisional surgical site infection occurred in two patients in each group and were treated on an outpatient basis with antibiotics. The mean length of hospitalization did not differ significantly between the Control and IMN groups [2.51 ± 2.02 vs. 2.32 ± 1.37 days, *p* = 0.79].

Regarding vaginal cuff-related complications, one patient in the control group developed a vaginal cuff hematoma on the postoperative 4th day and required hospitalization for conservative management. In the IMN group, one patient was hospitalized on the postoperative 7th day for vaginal cuff bleeding and underwent bleeding control with suturing under anesthesia. Additionally, two patients in the IMN group developed infectious vaginal cuff complications: one presented with a vaginal cuff abscess on postoperative day 10 and was hospitalized for antibiotic treatment and drainage, while another required hospitalization for vaginal cuff infection and received antibiotic therapy.

### 3.2. Vaginal Cuff Healing

**Figure 2:**
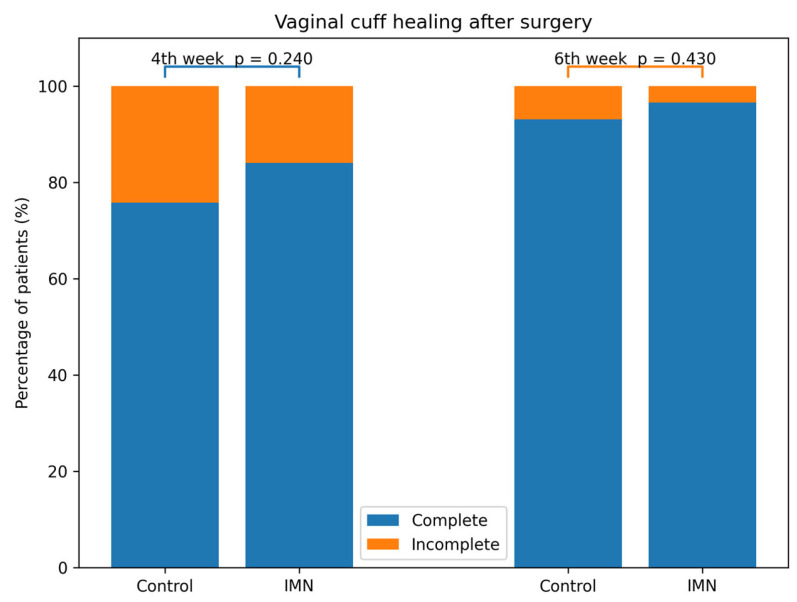
Graphical comparison of vaginal cuff healing scores at the postoperative 4th and 6th weeks between the groups.

At the postoperative 4th week, complete vaginal cuff healing was observed in 75.8% of patients in the control group and 84.1% in the IMN group [*p* = 0.24]. By the postoperative 6th week, complete vaginal cuff healing rates increased to 93.1% in the control group and 96.6% in the IMN group, with no statistically significant difference between groups [*p* = 0.43]. Graphical comparisons of vaginal cuff healing at postoperative weeks 4 and 6 are presented in Figure 2, and detailed numerical data are provided in Appendix A Table A1.

**Figure 3:**
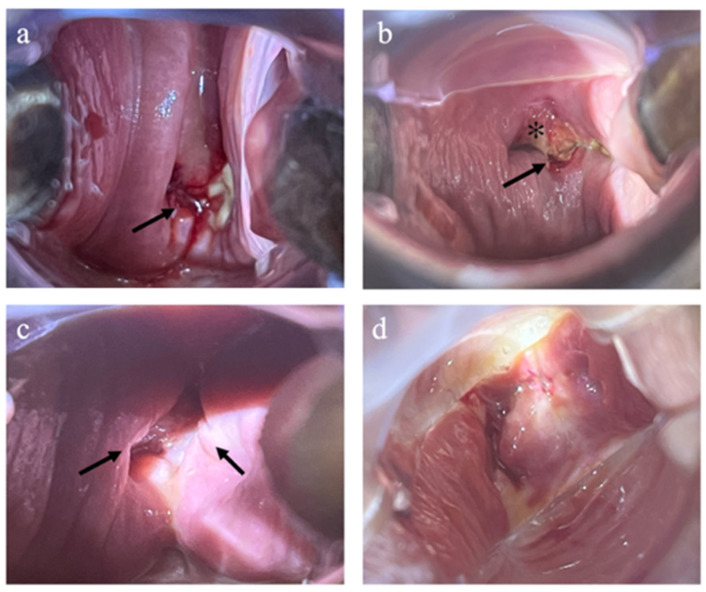
Representative images demonstrating incomplete and complete vaginal cuff healing. (**a**,**b**) Incomplete vaginal cuff healing. Arrows indicate areas of visible suture material and incomplete mucosal approximation. The asterisk in panel (**b**) denotes granulation tissue. (**c**) Localized vaginal cuff dehiscence; arrows indicate full-thickness separation of the vaginal epithelium. (**d**) Complete vaginal cuff healing, characterized by total mucosal approximation without mucosal separation and exposed suture.

In the IMN group, one patient demonstrated localized vaginal cuff dehiscence at the postoperative 4th week evaluation. As there was no evidence of evisceration and the patient remained asymptomatic, with benign final pathology, expectant management was adopted. At the postoperative 6th week follow-up, a reduction in the size of the dehiscence was observed. Representative examples of vaginal cuff assessment are shown in Figure 3.

**Figure A1:**
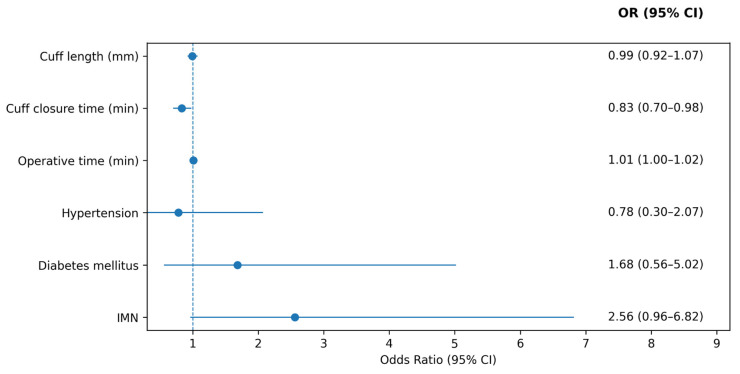
Multivariable logistic regression analysis of factors associated with complete vaginal cuff healing at the postoperative 4th week.

Univariable analyses assessed the association of postoperative IMN use and vaginal cuff closure time with complete vaginal cuff healing at the postoperative 4th week; neither variable showed a statistically significant association. In the multivariable logistic regression analysis, variables were selected a priori based on their potential influence on wound healing and included postoperative IMN, diabetes mellitus, hypertension, operative time, vaginal cuff closure time, and cuff length. Postoperative IMN was associated with higher likelihood of complete vaginal cuff healing at the postoperative 4th week [odds ratio [OR] 2.56, 95% confidence interval [CI] 0.96–6.82], but did not achieve statistical significance [*p* = 0.06]. Longer vaginal cuff closure time was independently associated with a lower probability of complete early vaginal cuff healing [*p* = 0.02] [Appendix A Figure A1]. Analyses were conducted according to the intention-to-treat principle.

### 3.3. Compliance and Tolerance

**Figure A2:**
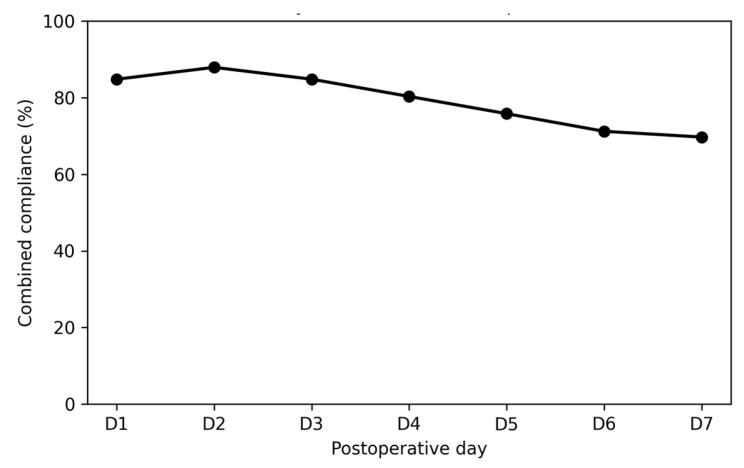
Day-level combined compliance with postoperative IMDs.

In the IMN group, the median combined compliance over postoperative days 1 to 7 was 100% [IQR 71–100], while the mean combined compliance was 79.2 ± 35.0%. A total of 48 out of 66 patients [72.7%] achieved good compliance [≥80%], and 43 out of 66 patients [65.2%] completed the full prescribed regimen [100%]. Combined compliance at the day level declined from 84.8% on the first postoperative day to 69.7% on the 7th postoperative day, indicating reduced adherence during the later postoperative period [Appendix A Figure A2].

IMDs were well tolerated, with 93.9% of patients completing the regimen. Incomplete intake of the prescribed regimen was observed in 6.1% of patients due to mild intolerance [diarrhea in one patient and taste aversion in three patients], and no severe adverse events were observed.

## 4. Discussion

In this prospective cohort study, patients who received immune-modulating diets (IMDs) had a higher rate of complete vaginal cuff healing at the 4th postoperative week than those on a standard diet; however, this difference did not reach statistical significance. By the 6th postoperative week, rates of complete vaginal cuff healing were high and comparable between the groups. The incidence of postoperative complications within 30 days was also similar.

Immunonutrition (IMN) has been demonstrated to modulate immune and inflammatory responses, enhance collagen synthesis, and reduce postoperative infectious complications in patients undergoing major oncologic surgery [6,12]. In alignment with this evidence, international guidelines recommend perioperative IMN for selected high-risk surgical populations [7]. However, evidence regarding the role of IMN in gynecologic oncology, particularly after laparoscopic surgery, remains limited.

In the present study, the daily IMN dose was consistent with regimens reported in the literature; however, supplementation was limited to the postoperative period due to clinical considerations. The elective laparoscopic approach and brief preoperative admission period in this cohort precluded standardized preoperative supplementation. As highlighted in the Enhanced Recovery After Surgery literature, barriers to routine-preoperative IMN in gynecologic oncology include limited preoperative patient contact, infrastructural constraints, cost considerations, and reduced patient compliance [13,14]. Evidence from six meta-analyses suggests that perioperative IMN provides the greatest benefit in surgical oncology, while postoperative administration alone confers a slightly reduced but comparable effect, and preoperative-only supplementation yields less benefit [15]. However, current guidelines provide limited direction regarding the optimal timing, duration, and dosage of IMN [16]. The absence of standardized recommendations regarding the optimal timing, duration, and dosage of IMN may partly explain heterogeneity across published studies and the lack of statistically significant findings in the present cohort.

Both study groups exhibited high rates of complete vaginal cuff healing at early postoperative time points. Previous cohorts have reported complete healing rates of approximately 80% at the 6th weeks and over 95% at the 8th weeks following open hysterectomy [17]. In contrast, the present cohort included patients with comorbidities known to impair wound healing, yet still demonstrated high rates of complete healing at the 4th and 6th postoperative weeks [18]. These results do not support concerns that laparoscopic colpotomy with electrosurgical energy inherently compromises vaginal cuff healing [19]. Instead, they suggest that surgeon experience and adherence to standardized, full-thickness vaginal cuff closure techniques may mitigate potential risks associated with energy use and magnification.

Vaginal cuff closure time emerged as an independent factor associated with early vaginal cuff healing. A longer closure duration may reflect increased technical difficulty, friable tissue quality, or greater local tissue trauma during suturing, all of which may impair microvascular perfusion and delay the early phases of wound repair. In contrast, cuff length was not independently associated with early healing outcomes, indicating that surgical technique and efficiency may be more influential than anatomical dimensions alone.

Although vaginal cuff healing was classified as complete according to the predefined clinical criteria in this study, it is important to recognize that complete mucosal healing at early postoperative assessment does not eliminate the risk of subsequent vaginal cuff dehiscence [20]. Since dehiscence can occur several months after hysterectomy, studies with extended follow-up are necessary to determine whether IMN confers protection against late vaginal cuff complications, particularly dehiscence, which is a rare but clinically significant adverse outcome following laparoscopic staging for gynecologic malignancies.

Postoperative compliance with IMDs was high, with approximately 88% of patients consuming at least 75% of the planned IMN dose. This finding contrasts with previously reported compliance rates, where adherence to IMN, particularly postoperatively, has been substantially lower. A recent narrative review of IMN within ERAS protocols for gynecologic oncology noted that postoperative compliance declined from nearly 78% in the preoperative period to approximately 28% after surgery, primarily due to nausea, anorexia, and impaired oral intake [16]. The high compliance observed in this cohort may be attributable to the minimally invasive surgical approach, which is associated with faster gastrointestinal recovery, earlier resumption of oral intake, and a lower incidence of postoperative nausea and vomiting. Similar improvements in tolerance and adherence have been reported in laparoscopic colorectal surgery when IMN is combined with minimally invasive surgical techniques [21]. The gradual decline in compliance observed during the later postoperative days may additionally reflect regimen fatigue, palatability issues, or reduced motivation as patients resumed a normal diet and daily activities. Future studies incorporating structured patient-reported feedback may help better identify barriers to adherence and optimize postoperative immunonutrition protocols.

Postoperative morbidity did not differ between the study groups. Although perioperative IMN has been shown to reduce hospital and intensive care unit length of stay in surgical populations requiring prolonged postoperative care, such as patients undergoing gastrointestinal, head and neck, or ovarian cancer surgery, these benefits may be less apparent in the context of laparoscopic staging for gynecologic malignancy, where postoperative recovery is inherently rapid, incisions are small, and hospital stay is already brief [22,23].

An additional methodological consideration involves a protocol modification implemented during the study period, in which the timing of vaginal cuff assessment was changed from the 6th to the 4th postoperative week. To maintain consistency in outcome measurement, patients who had already completed their initial evaluation at the 6th week were excluded from the primary analysis. Although this modification was applied uniformly and was not influenced by group-specific outcomes, changing the assessment time point during the study could have affected observed healing rates and introduced selection bias. Consequently, the results should be interpreted in light of this temporal adjustment in outcome measurement.

Several limitations warrant consideration. While baseline demographic, clinical, and surgical characteristics were comparable across groups, the observational and non-randomized design may have introduced selection bias and residual confounding. This risk is heightened because allocation to immunonutrition was determined by postoperative administration of immune-modulating diets rather than randomization. Additionally, no formal a priori sample size calculation was conducted, as this prospective observational study enrolled patients consecutively over a predefined period. Based on the observed difference in complete vaginal cuff healing at postoperative week 4 (86.2% vs. 74.6%), a post hoc calculation indicates that approximately 180 patients per group would be required to detect this difference with 80% power at a two-sided alpha level of 0.05. Consequently, the present cohort was likely underpowered to detect modest but clinically meaningful effects. The relatively wide confidence interval and borderline statistical significance further support this interpretation. Moreover, immunonutrition was administered exclusively in the postoperative period, precluding evaluation of potential benefits from preoperative or perioperative supplementation. Although multiple surgeons participated, the vaginal cuff closure technique and suture material were standardized to minimize inter-operator variability.

## 5. Conclusions

Postoperative IMN was not independently associated with early vaginal cuff healing following laparoscopic surgical staging for endometrial cancer. Surgical factors, particularly cuff closure time, appeared to be the primary determinants of vaginal cuff healing. The incidence of postoperative complications within 30 days was similar between patients receiving IMN and those managed with a standard postoperative diet. Further randomized studies evaluating standardized perioperative IMN protocols, with extended follow- up for late vaginal cuff complications, are recommended.

## Figures and Tables

**Table 1:** Baseline Characteristics.

Variable	Control (*n* = 62)	IMN (*n* = 69)	*p* Value
Age (years) (mean ± SD)	58.1 ± 11.0	56.4 ± 9.3	0.34
Gravida (median (IQR))	2 (0–10)	3 (0–8)	0.14
BMI ^1^ (kg/m^2^) (mean ± SD)	32 ± 8.2	31 ± 6.6	0.70
Smoking, *n* (%)	5 (8)	9 (13)	0.56
Active smoker	3 (4)	6 (9)	
Ex-smoker	2 (3)	3 (4)	
Menopausal status, *n* (%)			0.21
Postmenopausal	42 (68)	37 (53)	
Pre/perimenopausal	20 (32)	32 (46)	
Comorbid conditions, *n* (%)			0.64
Diabetes	29 (47)	39 (59)	
HbA1c ^2^ < 7.0	18 (62)	25 (64)	
HbA1c ≥ 7.0	11 (37)	14 (35)	
Hypertension	28 (45)	28 (40)	
Malignancy	7 (11)	5 (7.5)	
Breast	6 (85)	3 (60)	
Thyroid	1 (14)	1 (20)	
Colorectal	0 (0)	1 (20)	
ASA score ^3^, *n* (%)			0.55
ASA 2	35 (56)	33 (47)	
ASA 3	27 (44)	33 (47)	
ASA 4	0	3 (4)	
NRS score ^4^, *n* (%)			0.32
0	34 (55)	33 (47)	
1	25 (41)	27 (39)	
2	3 (4)	9 (14)	

Continuous variables are presented as mean ± standard deviation or median (range), as appropriate. Categorical variables are presented as number (%). ^1^ BMI, body mass index; ^2^ HbA1c, glycated hemoglobin (HbA1c < 7.0% was considered indicative of controlled diabetes); ^3^ ASA, American Society of Anesthesiologists physical status classification; NRS-2002, ^4^ Nutritional Risk Screening 2002 (a score < 3 was considered not at nutritional risk).

**Table 2:** Surgical and pathological characteristics.

Variable	Control (*n* = 62)	IMN (*n* = 69)	*p* Value
Operation time (min–max)	53 (30–215)	50 (28–212)	0.16
Cuff closure time (min–max)	12 (11–14)	12 (9–15)	0.93
Cuff length (mm)	42 (33–65)	45 (30–60)	0.27
Uterine weight (grams)	121.5 (47–375)	139.0 (58–420)	0.21
Procedure *n* (%)			0.67
TLH (+/−BSO) ^1^	44 (70)	53 (76)	
Staging	18 (30)	16 (24)	
FIGO stage ^2^ *n* (%)			0.76
Stage 1A1	-	2 (3)	
Stage 1A2	23 (37)	25 (37)	
Stage 1B	7 (11)	6 (8)	
Stage 2A	2 (3)	3 (4)	
Stage 2B	4 (6)	6 (9)	
Stage 2C	2 (3)	3 (4)	
Stage 3A1	1 (1)	1 (1)	
Stage 3C1	2 (3)	2 (3)	
Benign	21 (33)	21 (31)	
Histological type *n* (%)			0.25
Endometrioid	38 (61)	45 (65)	
Serous	2 (3)	1 (1)	
Clear cell	0	1 (1)	
MMMT ^3^	0	1 (1)	
Mixt Type	1 (1)	0	
EIN ^4^	3 (4)	8 (11)	
Endometrial hyperplasia	5 (8)	0	
Benign	13 (21)	12 (17)	

Continuous variables are presented as median (range). Categorical variables are presented as number (%).^1^ TLH, total laparoscopic hysterectomy; BSO, bilateral salpingo-oophorectomy; ^2^ FIGO, International Federation of Gynecology and Obstetrics (FIGO stage was defined according to the 2023 FIGO classification); ^3^ MMMT, malignant mixed Müllerian tumor; ^4^ EIN, endometrial intraepithelial neoplasia.

**Table 3:** Comparison of postoperative morbidity between groups.

Outcome	Control (*n* = 62)	IMN (*n* = 69)	*p* Value
Febrile morbidity, *n* (%)	2 (3)	1 (1)	0.62
Readmission, *n* (%)	1 (1)	3 (4)	0.36
SSI ^1^, *n* (%)			0.69
Abdominal incision	2 (3)	2 (3)	
Vaginal cuff infection	-	2 (3)	
Vaginal cuff bleeding, *n* (%)	1 (1)	1 (1)	1.00
LOS ^2^ (days), mean ± SD	2.51 ± 2.02	2.32 ± 1.37	0.79

^1^ SSI, surgical site infection; ^2^ LOS, length of hospital stay.

## Data Availability

The raw data supporting the conclusions of this article will be made available by the authors on request.

## Abbreviations

The following abbreviations are used in this manuscript:

CDC	Centers for Disease Control and Prevention
ESPEN	European Society for Clinical Nutrition and Metabolism
ERAS	Enhanced Recovery After Surgery
NRS	Nutritional Risk Screening

## Appendix A

**Table A1:** The incidence of complete vaginal cuff wound healing at the 4th and 6th weeks after operation.

Cuff Healing (Postoperative 4th Week)	Control *n* (%)	IMN *n* (%)	*p* Value
Total	47 (75)	58 (84)	0.24
Subtotal	15 (24)	11 (15)	
Total patients	62 (100)	69 (100)	
Cuff healing (Po 6th week)			
Total	53 (93)	56 (96)	0.43
Subtotal	4 (6)	2 (3)	
Total patients	58	59	
